# Cohort study of the effects of occupation and environmental tobacco smoke on the incidence of Alzheimer’s disease among seniors

**DOI:** 10.18332/tid/157208

**Published:** 2023-02-06

**Authors:** Li Yang, Wenjie Wan, Cheng Xuan, Caiyan Yu, Kailing Jin, Pinpin Zheng, Jing Yan

**Affiliations:** 1 Zhejiang Provincial Research Center for Prevention and Treatment of Cardiovascular and Cerebrovascular Diseases, Zhejiang Hospital, Hangzhou, China; 2 Key Laboratory of Public Health Safety, Ministry of Education, Health Communication Institute, Fudan University, Shanghai, China; 3 Fourth Clinical Medical College, Zhejiang Chinese Medical University, Hangzhou, China; 4 Chronic Disease Control Department, Zhuji City People's Hospital, Zhuji, China

**Keywords:** Alzheimer’s disease, environmental tobacco smoke, occupation, seniors

## Abstract

**INTRODUCTION:**

Alzheimer’s disease (AD) is a disease caused by many factors including occupational and environmental factors. Secondhand smoke (SHS) can affect cognitive function. However, there is limited recent epidemiological research on how SHS and occupational factors affect AD in Zhejiang province.

**METHODS:**

We established a cohort of an AD high-risk population. In 2018, a cohort of 1742 elderly aged ≥60 years was established. In 2020, the cohort was followed up, and a total of 1545 people participated in the two surveys. Data collection included demographic and economic information such as age, gender, occupation, education level etc., and relative health behavior information such as smoking, drinking and tea drinking, etc. Basic physical examination data included height, weight, blood pressure, etc. At the same time, related cognitive status was assessed. Cox proportional hazards regression models, both unadjusted and adjusted models, were used to determine associations between cohort characteristics and AD.

**RESULTS:**

The results showed that SHS exposure and occupational characteristics were associated with an increased risk of cognitive impairments in seniors. Subgroups who used to work as blue-collar workers, who never worked, who kept standing for most of the time at work, and who were engaged in hard physical labor prior to retirement, had high incidence rates of AD.

**CONCLUSIONS:**

It was revealed that SHS, standing for most of the time and hard physical labor were associated risk factors of AD among seniors, compared with white-collar work. We recommend that the government establish a community supervisory mechanism to persuade smokers to quit or control smoking.

## INTRODUCTION

Alzheimer’s disease (AD) is a chronic central nervous system disorder that develops progressively from an insidious onset. It is characterized by progressive memory impairment and cognitive loss, accompanied by a decreasing ability to live a normal life and behavioral changes. The incidence of this most common type of dementia increases with age. According to the *2020 Alzheimer’s Disease Facts and Figures*, about 50 million people in the world suffer from AD or other types of dementia, estimated to reach 152 million by 2050^1^. The National Institute on Aging found that AD is the third leading cause of death for seniors, second only to heart disease and cancer; in the United Kingdom, dementia has become the leading cause of death.

Mild cognitive impairment (MCI) has been identified as an intermediate stage between normal cognitive ageing and dementia. At present, in China, the MCI prevalence is up to 15.5% among people aged ≥60 years, and more than 15 million people suffer from dementia (including 9.83 million AD patients). More importantly, the Chinese population is aging, with about 180 million people aged >65 years in 2020, or about 13% of the total population. As the aging process gains momentum, the number of AD patients is growing, imposing a huge economic burden on the country^2^. Clearly, AD has developed into a health crisis for modern people, and effectively containing the continued high incidence of AD has become a huge challenge in this aging society.

AD is a disease caused by cumulative dangerous risks, among which both occupational and environmental factors play a part^3-5^. Those engaged in a highly professional job that needs high social interaction and attention-control skills can better cope with neuronal degeneration and thus maintain their cognitive function^3^. The concept of cognitive reserve (CR) suggests that the brain actively attempts to cope with brain damage by using pre-existing cognitive processing approaches or by enlisting compensatory approaches^4^. Social skills manifested by occupation were associated with severe hypo-metabolism in medial and dorsolateral prefrontal regions, and cognitive control in the left fronto-insular cortex, which may affect the cognitive function^3^.

Secondhand smoke (SHS) also referred to as passive smoking, refers to the exposure of non-smokers to mixed smoke released from tobacco products used by smokers^5^. SHS contains nicotine, the major contributor to the addictive properties of tobacco, and carbon monoxide, which affect the cardiovascular system. SHS exposure can damage the cardiovascular system by increasing platelet coagulation and lead to endothelial dysfunction^6,7^. Studies have shown that exposure to tobacco was positively correlated with cognitive impairment, dementia and other neurodegenerative diseases. Using the standard method of Geriatric Mental State Examination (GMS), Chen et al.^8^ interviewed 5921 people aged ≥60 years in five provinces in China in 2007–2009 and characterized their SHS exposure. Significant associations with severe dementia syndromes were found in never smokers and in former/current smokers. There were no positive associations between SHS and moderate dementia syndromes^8^. He et al.^3^ evaluated 7000 elderly residents from six regions within eastern China and the results showed that passive smoking exposure increased the risk of cognitive impairment in older adults, especially in non-smokers. In this study, we aimed to update the results in the field and conduct epidemiological research on how SHS and occupational factors affect AD in Zhejiang province.

## METHODS

### Population

We established a cohort of an AD high-risk population in Zhejiang Province. Inclusion criteria were: aged ≥60 years with normal cognitive function, permanent residents living in the selected community for more than half a year, and agreed to participate in the investigation and relevant referral. Exclusion criteria: other types of neurodegenerative diseases such as vascular cognitive impairment, dementia with Lewy bodies, fronto-temporal dementia, Parkinson’s disease, hippocampal sclerosis, hypothyroidism, vitamin B12 deficiency, using anticonvulsants, neuroleptics, antiemetic drugs, and acute critical disease.

In 2018, a cohort of AD high-risk population was established, and demographic data and blood samples were collected. The population aged ≥60 years in the selected area was considered the high-risk population for AD, as advanced age was considered the most important factor for AD. Twelve administrative districts were divided into four types of districts based on economic level. From each of these four groups, one district was systematically selected. Then one community was randomly chosen from each district. Subjects aged ≥60 years were invited to participate in the selected communities.

### Measures

Trained community nurses conducted the survey in community health service centers or at the participants’ residences. Data collection included questionnaires and basic physical examinations. The information in the questionnaire was obtained from the subjects after informed consent. Information collection included demographic and economic information such as age, gender, occupation, education level, etc. and relative health behavior information such as smoking, drinking and tea drinking, etc. Basic physical examination data included height, weight, blood pressure (BP), etc. At the same time, related cognitive status was assessed. Specifically trained psychiatrists conducted the diagnosis process based on guidelines (Guidelines group of Alzheimer’s Disease Branch of ADC, 2021), and combined with the results of the MMSE and MoCA^9,10^, magnetic resonance imaging (MRI) was used when needed.

Smoking was defined as continuous or cumulative smoking for 6 months or more, and this important information was collected by a questionnaire, that included the question: ‘How long have you been smoking continuously or cumulatively?’.

Participants were asked to report on the frequency of SHS exposure in their environment in the past year in the questionnaire survey. Frequency of SHS exposure was measured with the following question: ‘How often does X smoke when you are around?’, where X includes relatives, friends, colleagues and others, in their environment. Response items ranged from 0 to 8, more than 3 times a day. If relatives, colleagues or others did not smoke, a score of zero was assigned. Participants who were exposed to tobacco products 3 times or more a day were considered as SHS exposed. The workers were dichotomized into white-collar workers (managers, professionals, technicians, clerks, and sales workers) and blue-collar workers (crafts, machine operators, and assemblers). Occupation was referred to the primary lifetime occupation prior to retirement, in which most working life was spent.

### Quality control

When measuring height and weight, participants were asked to wear light clothing, and no shoes. Blood pressure was measured in a sitting position, resting for at least 5 minutes before measurement; blood pressure was measured twice on the right upper arm using a standard electronic sphygmomanometer (Omron HEM-7430). If the difference between the two readings was greater than 10 mmHg, a third measurement was taken, and the average of the last two readings was used. MMSE test, including of Chinese version, was confirmed to have good reliability and validity^11,12^.

### Statistical analysis

Epidata 3.0 was used for data entry, and SAS 9.4 was used for data management and analysis. Frequencies and percentages were used for categorial data, and mean and standard deviation description was used for numerical data, to study the sociodemographic characteristics and cognitive function of the subjects. T-test and chi-squared test were used to compare the statistical differences. A health association analysis method used with follow-up data in prospective cohort studies is the Cox proportional hazards regression model; the ratio of any two risk functions refers to the relative hazard HR. Firstly, the unadjusted regression model was used to evaluate the cohort’s associated risk factors related to AD. Then, the regression model was adjusted with age, gender, and education level, and finally, the risk factors were determined. Data were considered censored if death occurred by the time of the second follow-up. All analyses were conducted by bilateral significance test, and the significance level of hypothesis test was set to p<0.05.

## RESULTS

### Baseline information

In all, 3500 cases were collected utilizing TV and newspaper advertisements, and invalid data such as those missing important information, were removed, totaling 2072 cases; 330 patients diagnosed with dementia at first survey were removed, and the number of people entering the cohort was 1742. In 2020, the subjects of the cohort were followed up, and a total of 1545 people participated in the two surveys. There was no statistical differences in the main demographic characteristic variables between the analysis data set before removing dementia cases and the whole data set of the first survey (2072 and 3500) and the second follow-up (1742 and 1545), including age, sex, living condition, education level, and major occupational type. The follow-up rate was 88.69%. The baseline characteristics of the cohort are given in Table 1.

**Table 1:** Baseline characteristics of the study cohort (N=1545)

*Characteristics*	*Categories*	*Total (N=1545) n (%)*	*Male (N=739) n (%)*	*Female (N=806) n (%)*	*p*
**Age** (years), mean ± SD		68.18 ± 4.8	68.6 ± 4.9	67.7 ± 4.6	<0.001
**Age group** (years)	60< to ≤64	403 (26.1)	174 (23.5)	229 (28.4)	0.001
	64< to ≤69	528 (34.2)	243 (32.9)	285 (35.4)	
	69< to ≤74	436 (28.2)	214 (29.0)	222 (27.5)	
	74< to ≤85	178 (11.5)	108 (14.6)	70 (8.7)	
**Marital status**	Living alone	205 (13.6)	63 (8.8)	142 (18.0)	<0.001
	Cohabitation	1297 (86.4)	651 (91.2)	646 (82.0)	
**Education level**	Illiteracy	232 (15.0)	61 (8.3)	171 (21.2)	<0.001
	Primary school	910 (58.9)	433 (58.6)	477 (59.2)	
	Junior high school	350 (22.7)	215 (29.1)	135 (16.7)	
	Senior high school or higher	53 (3.4)	30 (4.0)	23 (2.9)	
**Occupation**	Farmer	739 (47.8)	688 (51.9)	51 (50.0)	<0.001
	Blue-collar worker	705 (45.6)	626 (24.5)	79 (22.1)	
	White-collar worker	101 (6.5)	95 (23.6)	6 (27.9)	
**Anxiety**	No	1360 (96.2)	634 (96.6)	726 (95.9)	0.486
	Yes	53 (3.8)	22 (3.4)	31 (4.1)	
**Depression**	No	1255 (81.3)	620 (83.9)	635 (79.0)	0.015
	Yes	288 (18.7)	119 (16.1)	169 (21.0)	
**Waistline**	Normal	488 (32.5)	298 (41.7)	190 (24.2)	<0.001
	Abnormal	1012 (67.5)	416 (58.3)	596 (75.8)	
**BMI** (kg/m^2^), mean ± SD		24.47 ± 3.03	24.42 ± 2.88	24.51 ± 3.16	0.548
**BMI group**	Normal	689 (45.9)	329 (46.1)	360 (45.8)	0.146
	Overweight	167 (11.1)	68 (9.5)	99 (12.6)	
	Obese	644 (42.9)	317 (44.4)	327 (41.6)	
**SBP** (mmHg), mean ± SD		150.7 ± 20.0	149.9 ± 19.3	151.4 ± 20.6	0.147
**SBP group**	<140	451 (30.0)	227 (31.8)	224 (28.5)	0.234
	140–180	927 (61.8)	435 (60.9)	492 (62.5)	
	≥180	123 (8.2)	52 (7.3)	71 (9.0)	
**DBP** (mmHg), mean ± SD		81.4 ± 10.7	83.2 ± 11.1	79.7 ± 10.1	<0.001
**DBP group**	<90	1184 (78.9)	519 (72.7)	665 (84.5)	<0.001
	<110	307 (20.5)	188 (26.3)	119 (15.1)	
	≥110	10 (0.7)	7 (1.0)	3 (0.4)	
**HDL group** (mmol/L)	≥1.0	75 (5.1)	32 (4.6)	43 (5.5)	0.477
	<1.0	1399 (94.9)	666 (95.4)	733 (94.5)	
**TG group** (mmol/L)	<1.7	1002 (70.1)	504 (76.1)	498 (64.9)	<0.001
	≥1.7	427 (29.9)	158 (23.9)	269 (35.1)	
**TC group** (mmol/L)	<5.7	1095 (73.1)	576 (80.8)	519 (66.1)	<0.001
	≥5.7	403 (26.9)	137 (19.2)	266 (33.9)	
**LDL group** (mmol/L)	<3.3	1234 (82.3)	628 (88.1)	606 (77.0)	<0.001
	≥3.3	266 (17.7)	85 (11.9)	181 (23.0)	
**History of hypertension**	Yes	1073 (71.5)	508 (71.1)	565 (71.8)	0.819
	No	428 (28.5)	206 (28.9)	222 (28.2)	
**History of myocardial infarction**	Yes	3 (0.2)	0 (0.0)	3 (0.4)	0.251
	No	1498 (99.8)	714 (100.0)	784 (99.6)	

### Analysis on subgroups in the cohort study

From 2018 to 2020, 1545 participants in an AD high-risk population cohort finished the first and second follow-up, and there were 136 new cases diagnosed as having AD on the second follow-up, with an annual incidence rate of about 4.4%. Based on those aged ≥60 years in the sixth demographic census of Zhejiang Province in 2010, the annual incidence rate was 2.4% after age and gender standardization. The results showed that higher incidence rates of AD were found among those who used to work as blue-collar workers, who kept standing for most of the time at work, and who were engaged in hard physical labor work before retirement (Table 2). Among the 1545 participants, 203 were found to be smokers in the two surveys. After excluding smoking cases, analysis on the AD incidence rates of different SHS exposure groups showed that people exposed SHS were more vulnerable to AD (Table 3).

**Table 2:** Incidence rates of subgroups by occupation in the study cohort (N=1545)

*Variable*	*Group*	*Two-year incidence rate %*	*Normal (N=1409) n*	*Dementia (N=136) n*	*p*
**Occupation before retirement**	Farmer	6.9	688	51	<0.001
	Blue-collar worker	11.2	626	79	
	White-collar worker	5.9	95	6	
**Employment**	Employed	9.8	272	29	<0.001
	Not employed	3.4	800	28	
	Never employed	19.0	337	79	
**Way of working 1**	Sitting for most of the time	4.5	276	13	<0.001
	Standing for most of the time	9.8	1133	123	
**Way of working 2**	Mainly common physical activities	6.4	1259	86	<0.001
	Mainly hard physical labor	25.0	150	50	

**Table 3:** AD incidence rates of SHS subgroups in the study cohort

*Variables*	*Categories*	*AD incidence rate %*	*Normal (N=1226) n (%)*	*Dementia (N=116) n (%)*	*p*
**SHS in home** (days/week)	0	7.9	1038 (84.7)	89 (77.2)	0.01
	1–4	5.6	44 (3.6)	5 (4.4)	
	5–7	1.6	144 (11.7)	21 (18.4)	
**SHS in the workplace** (days/week)	0	4.2	837 (68.3)	57 (49.3)	0.008
	1–4	3.0	262 (21.4)	40 (34.5)	
	5–7	1.4	127 (10.3)	19 (16.2)	

AD: Alzheimer’s disease. SHS: secondhand smoke.

### Analysis of the effects of occupation and SHS on AD among seniors

The AD diagnosis result in the second follow-up as a dependent variable and the baseline characteristics as independent variables, Cox regression was used to screen the associated risks factors of AD among seniors. A univariate regression model was used first, followed by a multivariate regression model used to adjust variables including age, gender, and educational degree. The results showed that standing for most of the time (HR=1.07; 95% CI: 1.02–4.19) and mainly hard physical labor (HR=1.47; 95% CI: 1.14–2.59) were associated risk factors of AD, and that the primary lifetime occupation as white-collar workers before retirement might be a protective factor compared with blue-collar workers (HR=0.59; 95% CI: 0.14–0.97). SHS exposure for 5–7 days a week, in the home and workplace, was associated with the incidence of AD among seniors (Table 4).

**Table 4:** Analysis on effects of occupational and SHS exposure on the AD incidence rates in the study cohort

*Variables*	*Univariate regression model HR (95% CI)*	*Multivariate regression model HR (95% CI)*
**Occupation before retirement** (Ref. Blue-collar worker)		
Farmer	0.72 (0.30–1.73)	1.59 (0.84–3.00)
White-collar worker	0.96 (0.46–2.04)	0.59 (0.14–0.97)
**Way of working 1** (Ref. Sitting for most of the time)		
Standing for most time	2.66 (1.38–5.15)	1.07 (1.02–4.19)
**Way of working 2** (Ref. Mainly common physical activities)		
Mainly hard physical labor	1.44 (1.27–1.72)	1.47 (1.14–2.59)
**SHS in homes** (days/week) (Ref. 0 days/week)		
1–4	0.58 (0.07–4.83)	0.30 (0.02–3.77)
5–7	0.72 (0.48–1.10)	1.72 (1.48–2.10)
**SHS in the workplace** (days/week) (Ref. 0 days/week)		
1–4	0.92 (0.32–2.60)	0.49 (0.13–1.80)
5–7	0.82 (0.24–1.30)	1.21 (1.00–2.50)

AD: Alzheimer’s disease. SHS: secondhand smoke.

## DISCUSSION

Studies have shown that exposure to tobacco was positively correlated with cognitive impairments, dementia and other neurodegenerative diseases^13^, and likewise, passive smoking increased the risk of cognitive impairments or dementia^14-16^. Our findings show that there is an associated relationship between SHS exposure and increased risk of AD in seniors, for the elderly population exposed to SHS in home and workplace, for 5–7 days a week particularly, who were more likely to suffer from AD. Tobacco specific carcinogens might reduce the mass of neurons in specific areas of the brain that were related to learning and memory^11^. Some researchers included a dose-response test on SHS exposure and observed a risk trend between exposure dose and cognitive impairments^17^. Although some studies did not show an association between SHS exposure and dementia^5^, others showed that there was an inverse relationship between the cotinine level in serum and cognitive functions^11^, which support our results. These conflicting results might be related to individual variations in, for example, ventilation degrees in passive smokers’ living environments and effective doses for passive smoking exposure, insufficient sample size, and biased memories of participants.

Occupational activities could regulate the occurrence and clinical manifestations of the disease and serve as a substitute indicator for CR. This concept had been introduced to explain the mismatch between the severity of brain dysfunction and its clinical manifestations by showing that people with higher CR could cope with neuronal dysfunction better ^4^. In this study, subgroups who used to work as blue-collar workers, kept standing for most of the time at work, and engaged in hard physical labor, had high AD prevalence. Work that demanded excellent social skills and attentiveness control led to less reductions in the metabolism of the prefrontal cortex and insula^18^. The occupation was a premorbid feature that affected CR in dementia as it was involved in the planning^19^, episodic memory^20^ and executive abilities^21^, and was closely related to fronto-temporal neurodegeneration.

Patients’ better social skills and adaptability to the working environment are related to social cognitive abilities because they play a key role in internal monitoring actions^22^, adopting ideas^23^, learning theories of mind^24^ and showing compassion for the suffering of others^25^. Studies showed that, compared with white-collar workers, subjects who were engaged in physical labor were more vulnerable to cognitive impairments and AD^16^, which was consistent with our results. Jobs that required high intelligence helped people better cope with possible cognitive impairments in life.

### Limitations

Some limitations of this study need to be mentioned. The main limitation of the study was that the follow-up time for AD was relatively short. Also, there were no factors accounted for in particular occupation groups, e.g. pesticides in farmers and other chemicals in blue-collar workers, and the smoking status was unadjusted. Participants could be in both categories (in home or workplace) but we chose the main exposure style of SHS as the result in the table, which might had confounding effects on the results. As there was no evaluation of the dose-response relationship between SHS exposure and AD, it was not clear to what extent the effects of passive smoking on cognition were due to late-life exposure or exposure earlier in life. Finally, there still needs to be a good rationale for how the SHS and occupation might interrelate or interact, and whether SHS and workplace have synergistic effects, etc. All of these would need to be considered in future studies.

## CONCLUSIONS

SHS was associated with AD incidence rates, especially for workers with special occupations such as blue-collar workers, who kept standing for most of the time at work, and who were engaged in hard physical labor work. Early prevention measures of AD should be strengthened among these populations to prevent and delay the occurrence and development of AD. At the same time, considering that a majority of the population worldwide is not fully protected by smoke-free public health regulations, we call on families and societies to quit or reduce smoking, and we also recommend that the government establish a community supervisory mechanism to persuade smokers to quit or control smoking.

## Data Availability

The data supporting this research are available from the authors on reasonable request.
